# Cleavage of periostin by MMP9 protects mice from kidney cystic disease

**DOI:** 10.1371/journal.pone.0294922

**Published:** 2023-12-01

**Authors:** Nabila Djaziri, Cindy Burel, Lilia Abbad, Zeineb Bakey, Rémi Piedagnel, Brigitte Lelongt

**Affiliations:** 1 Sorbonne Université, Paris, France; 2 Institut National de la Santé et de la Recherche Médicale (INSERM) Unité Mixte de Recherche (UMR), Paris, France; Boston University School of Medicine, UNITED STATES

## Abstract

The matrix metalloproteinase MMP9 influences cellular morphology and function, and plays important roles in organogenesis and disease. It exerts both protective and deleterious effects in renal pathology, depending upon its specific substrates. To explore new functions for MMP9 in kidney cysts formation and disease progression, we generated a mouse model by breeding juvenile cystic kidney (jck) mice with MMP9 deficient mice. Specifically, we provide evidence that MMP9 is overexpressed in cystic tissue where its enzymatic activity is increased 7-fold. MMP9 deficiency in cystic kidney worsen cystic kidney diseases by decreasing renal function, favoring cyst expansion and fibrosis. In addition, we find that periostin is a new critical substrate for MMP9 and in its absence periostin accumulates in cystic lining cells. As periostin promotes renal cyst growth and interstitial fibrosis in polycystic kidney diseases, we propose that the control of periostin by MMP9 and its associated intracellular signaling pathways including integrins, integrin-linked kinase and focal adhesion kinase confers to MMP9 a protective effect on the severity of the disease.

## Introduction

There is a large variety of cystic kidney diseases including polycystic kidney disease (PKD), nephronophthisis (NPHP) and renal cystic dysplasia that differ in mode of inheritance, age of onset and clinical manifestation. PKD is characterized by progressive dilation of renal tubules that forms multiple fluid filled cysts and is the most common genetic disease that leads to kidney failure in humans [1, 2]. NPHP is a rare autosomal recessive genetically heterogeneous disorder, but it is a major genetic cause of end stage renal disease before adulthood. Characteristic pathological features are chronic tubulointerstitial nephritis, massive interstitial fibrosis and development of cortico-medullary cysts [3, 4].

Cysts results from important proliferation and apoptosis of tubular cells associated with an abnormal extracellular matrix remodeling [5], suggesting a role of metalloproteinases. Members of two families of enzymes, matrix metalloproteinases (MMPs) and A Disintegrin and Metalloproteinase (ADAM) might be involved in the development ok PKD as increased expression and activation of these enzymes, such as ADAM17 [6] and MMP2 [7] are observed in cystic collecting duct cells of Ift88 deficient mice and in cpk cystic mouse model, respectively. However, their specific role on cyst expansion is not fully understood. While inhibition of ADAM10 blocked the cystogenesis induced by polycystin1 knock-down in MDCK cells [8], the impact of MMPs inhibition on cyst formation is still unresolved. For instance, unspecific inhibition of MMPs family by doxycycline accelerates renal cyst growth and increases renal fibrosis in pcy mice [9] while it markedly inhibits the cystic disease progression in PCK rats [10]. Likewise, the treatment of cy/+ rats with the broad metalloproteinase inhibitor batimastat, resulted in a significant reduction of cyst number [11]. Besides, the effect of MMPs on cystic progression will be clarified by estimating the specific role of each distinct member of the MMPs’ family of enzymes. Among them, MMP9 is particularly interesting because of its variety of substrates (in addition to its principal substrate, type IV collagen) and its ability to degrade or activate other membrane molecules or non-matrix components [12–14]. Surprisingly, while increased level of protein expression or enzymatic activity is demonstrated in human serum [15], in human cystic tissue [10, 16] and in culture media of cells from rodent models of ADPKD [17], the role of MMP9 in cystic kidney disease is still not elucidated.

Here, we used the jck (juvenile cystic kidneys) mouse model which results from a double point mutation in the *Nek8* gene [18] to study the role of MMP9 in cyst progression. To this aim, we generate double mutant jck-MMP9 deficient mice on the same genetic background. They developed a more severe form of cystic disease than their wild-type mates, with exacerbated renal lesions, increased percentage of fibrosis and reduced life span. In search of new substrate of MMP9 which could be responsible of this protective effect, we uncover that MMP9 is able to degrade periostin. Elevated levels of periostin and β1 integrin in jck-MMP9 deficient mice are associated with integrin-linked kinase (ILK) and focal adhesion kinase (FAK) activation that are known to facilitate deleterious cystic phenotype by interfering with cell proliferation and cytoskeletal actin organization.

## Materials and methods

### Antibodies and recombinant proteins

Rabbit polyclonal antibody directed against anti-type IV collagen (20451) was from Novotec, (Bron, France). Mouse monoclonal anti-acetylated α-tubulin (T6199), antibody was purchased from Sigma Aldrich (Saint Louis, MI, USA), the monoclonal anti-PCNA, clone PC10 (M087901) from Agilent (Santa Clara, CA, USA), rabbit polyclonal anti-phospho FAK (Tyr397) was from Cell Signaling Technology (Danvers, MA, USA). Mouse monoclonal anti-FAK and rabbit polyclonal anti-β1 integrin antibodies were from Santa Cruz (Santa Cruz Biotechnology, Inc, Dallas, TX, USA), rat monoclonal anti-periostin antibody was from R&D Systems (Mineapolis, MN, USA), and polyclonal rabbit anti-αV integrin, anti- β3 integrin, and anti-ILK antibodies were from Proteintech Group, Inc (Rosement, IL, USA). Rat anti-Ki67 conjugated to eFluor^™^ 660, anti-rabbit, anti-mouse and anti-rat antibodies conjugated to Alexa Fluor 488, and 594 were obtained from Invitrogen (Thermo Fisher Scientific, Waltham, MA, USA). Anti-rabbit, anti-mouse and anti-rat conjugated to peroxidase and Dilochos Difluorus Agglutinin (DBA) lectin conjugated to rhodamine were purchased from Vector Laboratories (Burlingame, CA, USA). Recombinant mouse MMP9 was from Sino Biological (Eschborn, Germany) and recombinant mouse periostin from R&D Systems (Mineapolis, MN, USA).

### Mice

C57BL/6J-*Nek8* ^*jck*^ mice (jck-MMP9+/+) (Jackson laboratory, Bar habor, ME, USA) were bred with C57BL/6J-*Mmp9*^*tm1Tvu*^ mice [19] to generate C57BL/6J-*Nek8* ^*jck*^-*Mmp9*^*tm1Tvu*^ mice (jck-MMP9-/-) and were used after 15 backcrosses to C57BL/6J background. The mice were maintained with a 12 h light/12 h dark cycle and had free access to food and water. To prevent animal pain, we evaluate the progression of the disease by controlling daily mouse behavior and every 2 days body weight change. Mice that could not move properly or lose 10% body weight were sacrificed. At the end of the procedure, mice were killed by an intraperitoneal injection of an overdose of Euthasol vet (140 mg/kg). Mouse studies followed the Institutional Animal Care and Use Guidelines, and all protocols were approved by the *"Ministère de l’Education Nationale*, *de l’Enseignement Supérieur et de la Recherche"* (agreement number 00516.02).

### Periostin cleavage

To evaluate periostin cleavage by MMP9, 500 ng of recombinant mouse MMP9 tagged with 6His was activated with 1 mM 4-aminophenylmercuric acetate (APMA, Sigma Saint Louis, Mo, USA) and incubated at 37°C for 3 h with 50 ng of mouse recombinant periostin. Control experiment consisted of incubation with non-activated MMP9 (absence of MMP9 activation by APMA, proMMP9).

### Cell culture and proliferation

Mouse inner medullary collecting duct cell line 3 (IMCD-3) was cultured in DMEM:HAM’s F12 (1:1) (Thermo Fisher Scientific, Waltham, MA, USA) medium supplemented with 2 mM glutamine and 10% fetal calf serum (FCS). At 50% of confluency, FCS was removed for 48h and cells were incubated overnight in culture medium supplemented with activated (see above) recombinant mouse MMP9 (300 ng), recombinant mouse periostin (30 ng), and 30 ng of recombinant periostin preincubated with 300 ng of activated MMP9 (see above). Control consist of culture medium devoid of periostin and MMP9 and supplemented with APMA. The percentage of cell proliferation was determined on 5 photographs taken from 3 different experiments and analyzed with ImageJ software.

### Histology and analysis of fibrosis

Renal tissue was fixed in formalin and paraffin embedded. Three-micrometer–thick sections were stained with hematoxylin-eosin. Interstitial fibrosis was assessed on sirius red-stained paraffin sections (20 X) under polarized light on 8 different cystic jck-MMP9+/+ kidneys and jck-MMP9-/- scanned whole kidneys. It was quantified using computer-based morphometric analysis software (Axioplan, Axiovision, Zeiss, Germany). Data are expressed as percentage of positive areas examined.

Determination of total number of cyst and the percentage of cyst per surface area were performed on pictures of a whole kidney transversally sectioned and stained with hematoxilin/eosin. Microphotographs from 7 jck-MMP9-/- and 7 jck-MMP9+/+ kidneys were analyzed with NIH ImageJ software.

### Immunohistochemistry and immunofluorescence

Kidneys were transversally sectioned, fixed with 4% paraformaldehyde overnight at 4°C and embedded in paraffin. Antigen was unmasked on 5 μm sections by proteinase K (20 μg/mL) treatment for 20 min at room temperature, followed by incubation in 3% BSA in PBS for 30 min. Primary antibodies were diluted according to manufacturer’s instruction and applied overnight at 4°C followed by incubation with the second antibodies conjugated to Alexa Fluor (1/1000) for 1h 30 at room temperature. Slides were mounted in Permafluor and viewed under an Olympus IX83 microscope and confocal Zeiss 980 upright microscope and analysed with Image J software.

### Apoptose and proliferation in tissues

TUNEL (TdT mediated dUTP Nick End Labeling) method (Apoptag, QBiogene, Irvine, CA, USA) was performed according to the manufacturer’s instruction on 3 μm dewaxed and rehydrated sections. Kidney sections were counterstained with an antibody directed against Proliferating Cell Nuclear Antigen (PCNA, clone PC10, DakoCytomation SAS Glostrup, Denmark) conjugated to horseradish peroxidase HRP (for 90 min at room temperature) to detect proliferative cells. Sections were counterstained with DAPI (2 μg/mL for 30 s at room temperature) and viewed under a Zeiss Axiophot microscope (Carl Zeiss S.A., Le Pecq, France). The percentage of apoptotic nuclei and proliferative cells was determined on 6 microphotographs taken from each of 8 jck-MMP9-/- and 8 jck-MMP9+/+ kidneys sampled at 1 month and 4 months after birth.

### RNA extraction and quantitative real-time PCR

Total RNA was isolated with RNeasy Mini Kit (Qiagen, Hilden Germany) and eluted with RNAse free water. RNA quality was verified using a NanoDrop. 0.5μg RNA from 4 controls, 4 jck-MMP9+/+ and 4 jck-MMP9-/- kidneys was reverse transcribed to cDNA with PrimeScript^™^ RT reagent kit using manufacturer’s instructions (Takara Bio Inc., Kustsu, Japan). Real time PCR was performed with CFX96 Touch Real-Time PCR Detection System (BioRad, Hercules, California, USA) using SYBR Green PCR master Mix (Roche Diagnostics, Indianapolis, IN, USA). Specific primers for *Periostin* (forward primer: 5’-GCTGGCTGGAAAACAGCAAA-3’. Reverse primer: 5’-GAGGTCGCTAAGGCCAACTT-3’) and *Mmp9* cDNAs (forward primer: 5’-GCATACTTGTACCGCTATGG-3’. Reverse primer: 5’-TAACCGGAGGTGCAAACTGG-3’) were used for amplification at 95°C for 15 min, 45 cycles at 95°C for 15 s and 60°C for 15 s, and 72°C for 15 s. For quantitative analysis, *Periostin* and *Mmp9* mRNA expression was normalized to *Hprt* mRNA expression using the ΔΔCT method (forward primer: 5’-GGAGCGGTAGCACCTCCT-3’. Reverse primer: 5’-CTGGTTCATCATCGCTAATCAC-3’). Dissociation curves were analysed to determine that a single product was amplified.

### MMP9 activity by zymography

The gelatinolytic activity of MMP9 was demonstrated on tissue lysates and on cystic fluids from 6 control and cystic mice by zymography as previously described [20] on 50 μg of proteins. The presence of metalloproteinases was indicated by an unstained proteolytic zone of the substrate. Zymograms were analysed with Image J (National Institute of health, Bethesda, MD, USA) and converted to a graphical format.

### G/F actin determination

The G/F actin ratio was evaluated with the G-actin/F-actin In Vivo Assay kit according to the manufacturer’s instructions (Cytoskeleton Inc, Denver, Co, USA) in 4 jck-MMP9+/+ and 4 jck-MMP9-/- kidney cell lysates.

### Protein extraction and western blotting

Cells were lysed in 50 mM Tris-Hcl pH 7.5, 150 mM NaCl, 1% NP40, 0.5% deoxycholate and proteases inhibitors. Western blots were performed with 50 μg of protein lysates. Proteins were submitted to SDS-PAGE under reducing conditions in a 8 to 12% polyacrylamide gel and electro-transferred to polyvinylidene fluoride (PVDF) membrane (Millipore, St Quentin en Yvelines, France), blocked with 4% BSA for 30 min and incubated overnight at 4°C with primary antibodies. Secondary antibodies were conjugated to peroxidase (0.05 μg/mL), revealed with ECL Pico substrate (Expedeon, Cambridgeshire, United Kingdom) and detected with a gel imaging system ChemiDoc (BioRad, Hercules, California, USA).

### Statistics

Results are expressed as mean ± SEM. Statistical analysis were performed using GraphPad Prism software. Hypothesis tests were two-side. Significances of differences between different groups of mice were determined by ANOVA or t-test.

## Results

### MMP9 activity is increased in Nek8^jck^ (jck) kidney

**Fig 1:**
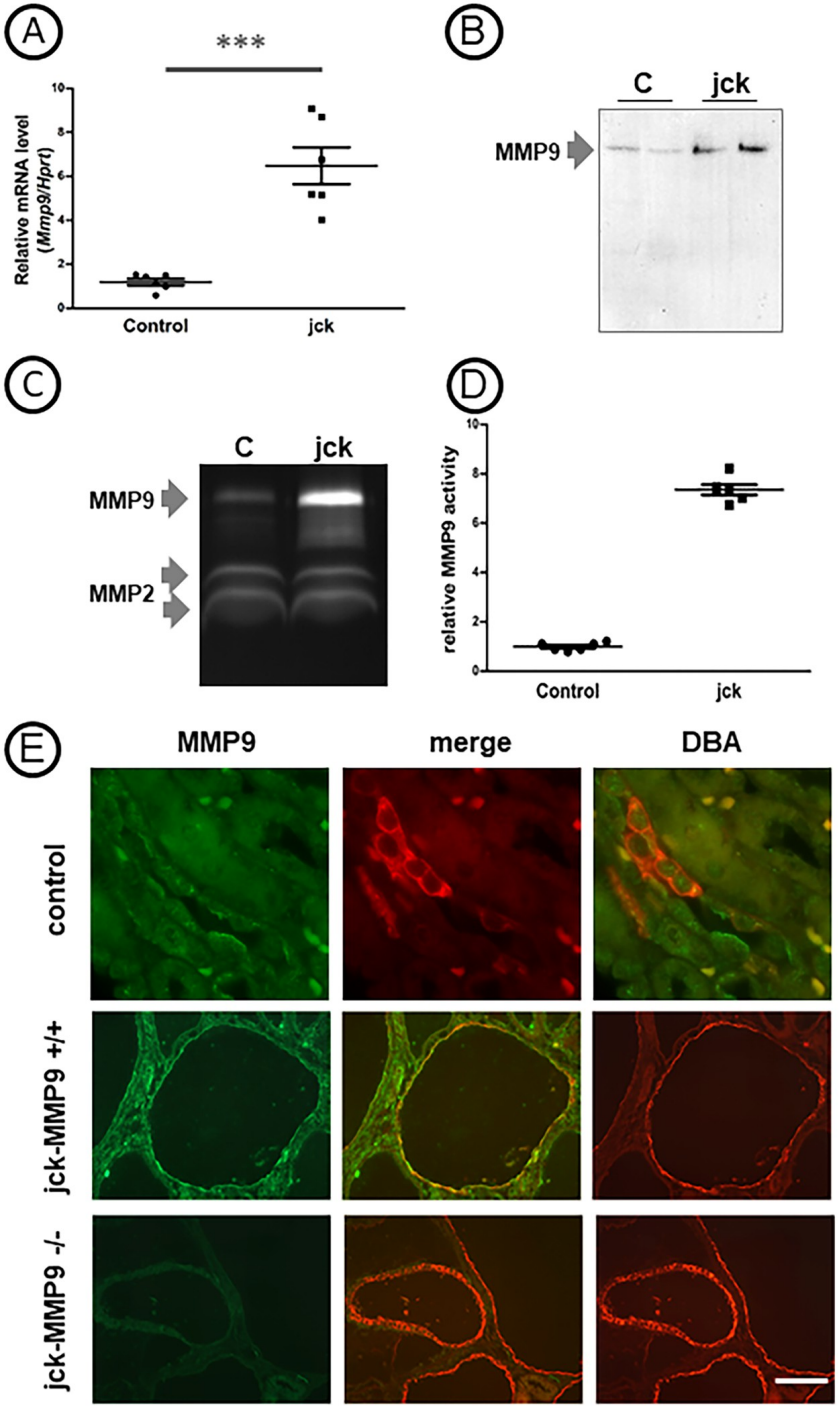
Expression of MMP9 in control and cystic Nek8^jck^ (jck) kidneys. (A) Quantitative real time RT-PCR determination of *Mmp9* gene expression normalized to *Haptoglobin-related protein* (*Hptr*) in 6 control C57bl6J and 6 jck kidneys. Note that *Mmp9* expression is significantly increased in jck-MMP9+/+ kidneys compared to controls. Values are mean ± SEM, ***p < 0.0001. (B) MMP9 protein level by representative Western blot of control C57bl6J and jck kidneys (C, D) MMP9 and MMP2 enzymatic activities assessed by zymography in tissue. (C) Representative zymogram shows that MMP9 activity is faint in control tissue lysates and dramatically increased in cystic kidneys from jck mice. Please note that level of expression of the active (upper band) and inactive (lower band) forms of MMP2 are not modified in cystic kidneys (jck). (D) Quantitative analysis of zymograms performed with protein lysates of 6 control and 6 jck kidneys Values are mean ± SEM, ***p < 0.0001. (E) Representative photographs of C57bl6J control (control) cystic control (jck-MMP9+/+) and MMP9 deficient (jck-MMP9-/-) kidneys stained with MMP9 revealed by Alexa Fluor 488 in green. MMP9 expression is observed in cysts derived from the collecting ducts stained with DBA lectin. Scale bar: upper panel: 5 μm; middle and lower panel: 100 μm.

We first compared the amount of MMP9 in tissue of control and of cystic jck-MMP9+/+ (jck) kidneys. We observed a ~7-fold increase of *Mmp9* mRNA by RT-qPCR (Fig 1A). This was associated with an increase level of the protein by Western blotting which was barely observed in control kidneys (c) and was dramatically enhanced in cystic kidneys (jck) (Fig 1B). MMP9 enzymatic activity monitored by zymography phenocopied the increase of MMP9 protein level in jck kidney (Fig 1C) and scanning of the zymograms showed a 6.8-fold increase (Fig 1D) of MMP9 enzymatic activity in jck cystic kidneys compared to controls. The expression of the active and inactive forms of MMP2, the other gelatinase present in kidney, was not changed (Fig 1C), suggesting a specific role of MMP9 in this rodent model of cystic disease. Immunostaining of MMP9 on paraffin kidney sections was observed in collecting ducts of control C57Bl6J kidneys (Fig 1E, upper panel), as previously shown in normal rabbit kidney [20] and in collecting ducts of jck-MMP9+/+ kidneys (Fig 1E, middle panel).

### MMP9 deficiency worsens cystic kidney disease

**Fig 2:**
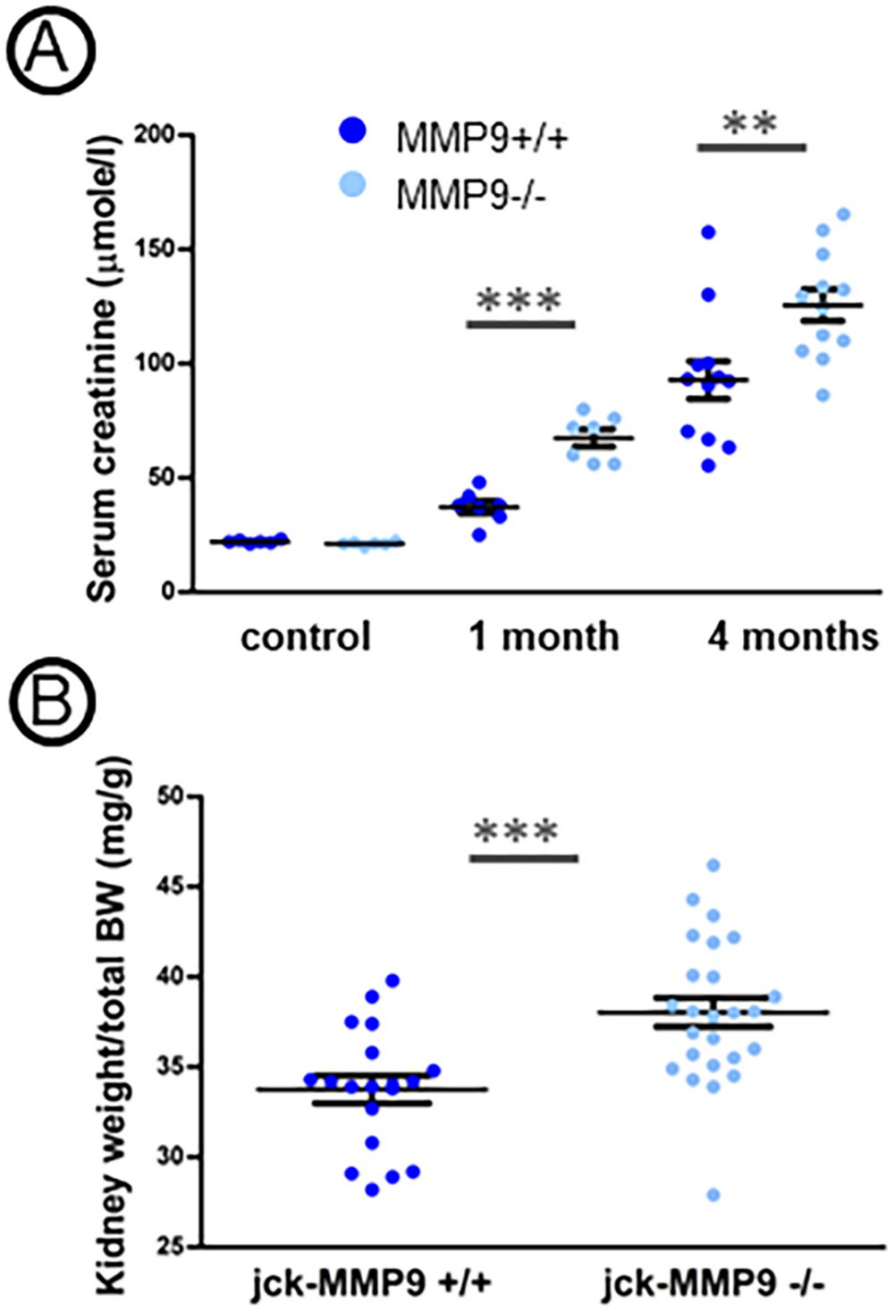
Effect of MMP9 on renal function and kidney weight. (A) Serum creatinine level in jck-MMP9+/+ and in jck-MMP9-/- at 1 month and at 4 months and in control C57bl6 mice aged 4 months (control). (B) Body weight to kidney weight ratio in 7 jck-MMP9+/+ mice and in 7 jck-MMP9-/- mice at 4 months. Note that the body weight to kidney weight ratio is already significantly increased in jck-MMP9-/- mice compared to control mice. Values are mean ± SEM, ***p < 0.0001, **p = 0.0059.

As expected, both jck-MMP9+/+ and jck-MMP9-/- polycystic mice developed renal failure but it was more pronounced in jck-MMP9 deficient mice, as attested by the significantly higher serum creatinine values in 1- and 4- month old jck-MMP9-/- mice compared to their jck-MMP9+/+ littermates (Fig 2A). The kidney weight normalized to body weight values was augmented in jck-MMP9-/- mice compared to jck-MMP9+/+ mice (Fig 2B).

**Fig 3:**
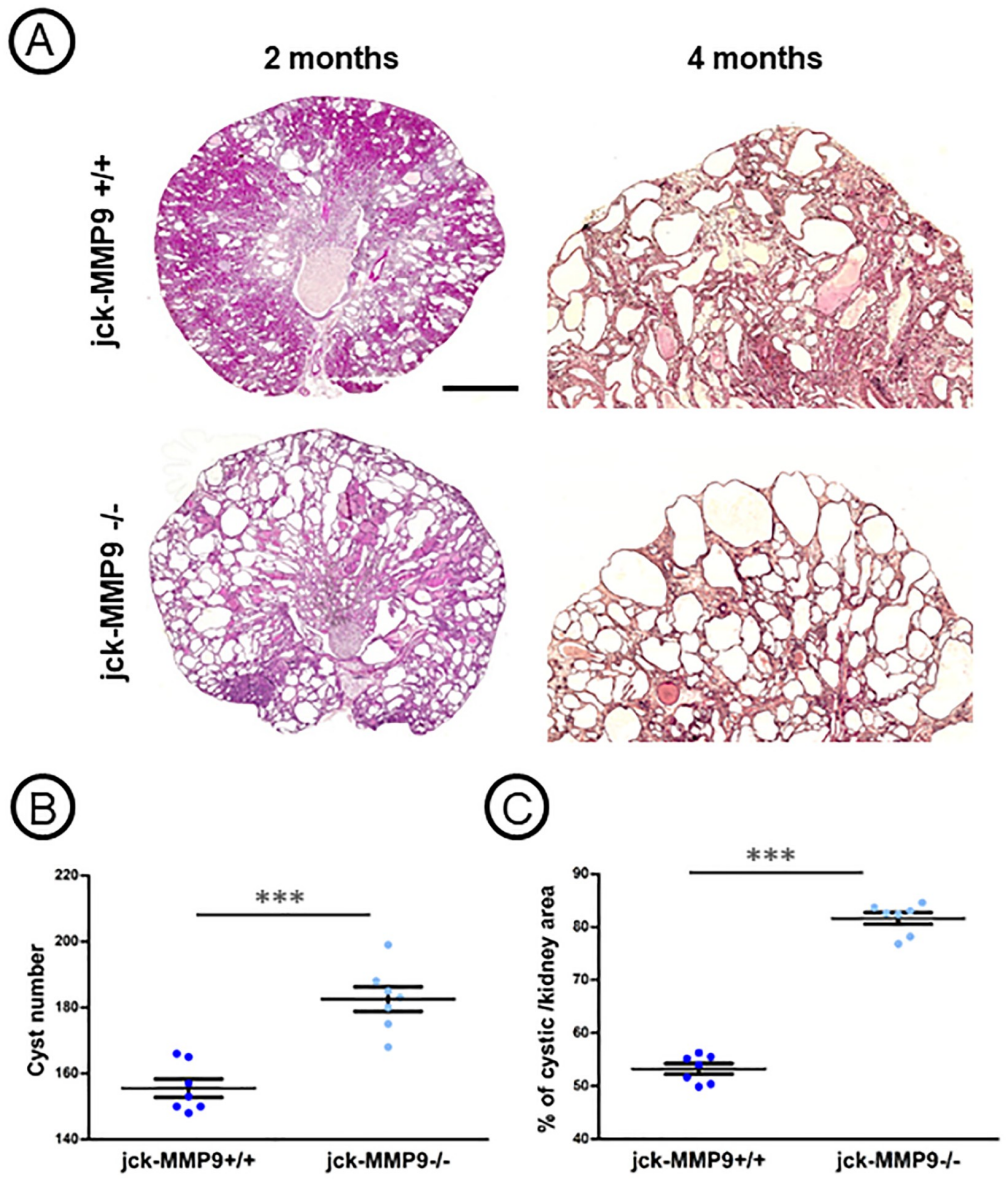
Kidney phenotype of jck-MMP9+/+ and in jck-MMP9-/- mice. (A) Representative microphotographs at 2 months and 4 months show that cysts appear greater in MMP9-/- cystic kidneys, scale bar: 1,100 μm at 2 months, and 250 μm at 4 months. (B, C) Effect of MMP9 on cyst number (B) and on the percentage of cystic area per total cross-sectional surface area at 4 months (C). These parameters were evaluated on 7 kidneys of each group. Cyst number and cyst percentage are significantly increased in kidneys of jck-MMP9-/- mice compared to control jck-MMP9. Values are mean ± SEM, ***p < 0.0001, **p = 0.0023.

**Fig 4:**
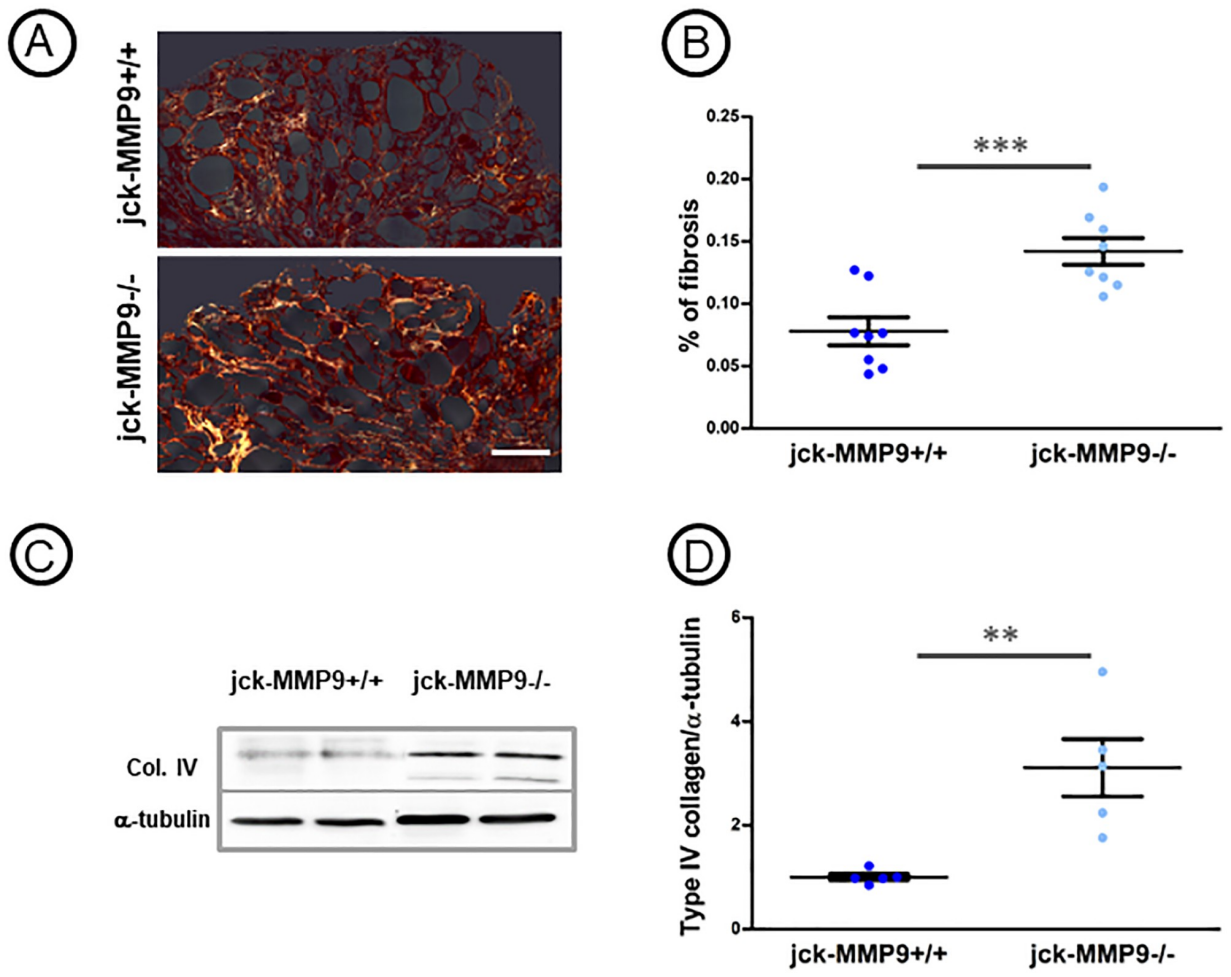
Effect of MMP9 on kidney fibrosis and type IV collagen expression. (A) Microphotographs of representative paraffin kidney sections of cystic jck-MMP9+/+ and jck-MMP9-/- kidneys stained with sirius red, scale bar: 250 μm. (B) Quantitative analysis of 8 different cystic jck-MMP9+/+ kidneys and jck-MMP9-/- kidneys. The percentage of fibrosis is significantly increased in jck-MMP9-/- compared to control jck-MMP9+/+ kidneys. (C) Representative western blot and (D) quantitative analysis performed on western blots with proteins lysates from 5 jck-MMP9+/+ and 5 jck-MMP9-/- kidneys. Note the significant increased expression of type IV collagen normalized to α-tubulin in jck-MMP9-/- mice. Values are mean ± SEM, ***p < 0.0001; **p = 0.0053. Scale bar: 100 μm.

Decreased renal function in jck-MMP9-/- was due to the greater severity of renal lesions observed on histological sections (Fig 3A) as shown by an increase of the number of cysts (Fig 3B) and of the percentage of cystic kidney tissue in jck-MMP9 -/- compared to jck-MMP9+/+ mice (Fig 3C). These histological lesions were associated with a greater extent of interstitial fibrosis at the end stage renal disease at 4 months (Fig 4A and 4B) and an increased expression of type IV collagen (Fig 4C and 4D).

### Periostin mRNA level is increased in Nek8^jck^ (jck) kidney but is not modified by MMP9

**Fig 5:**
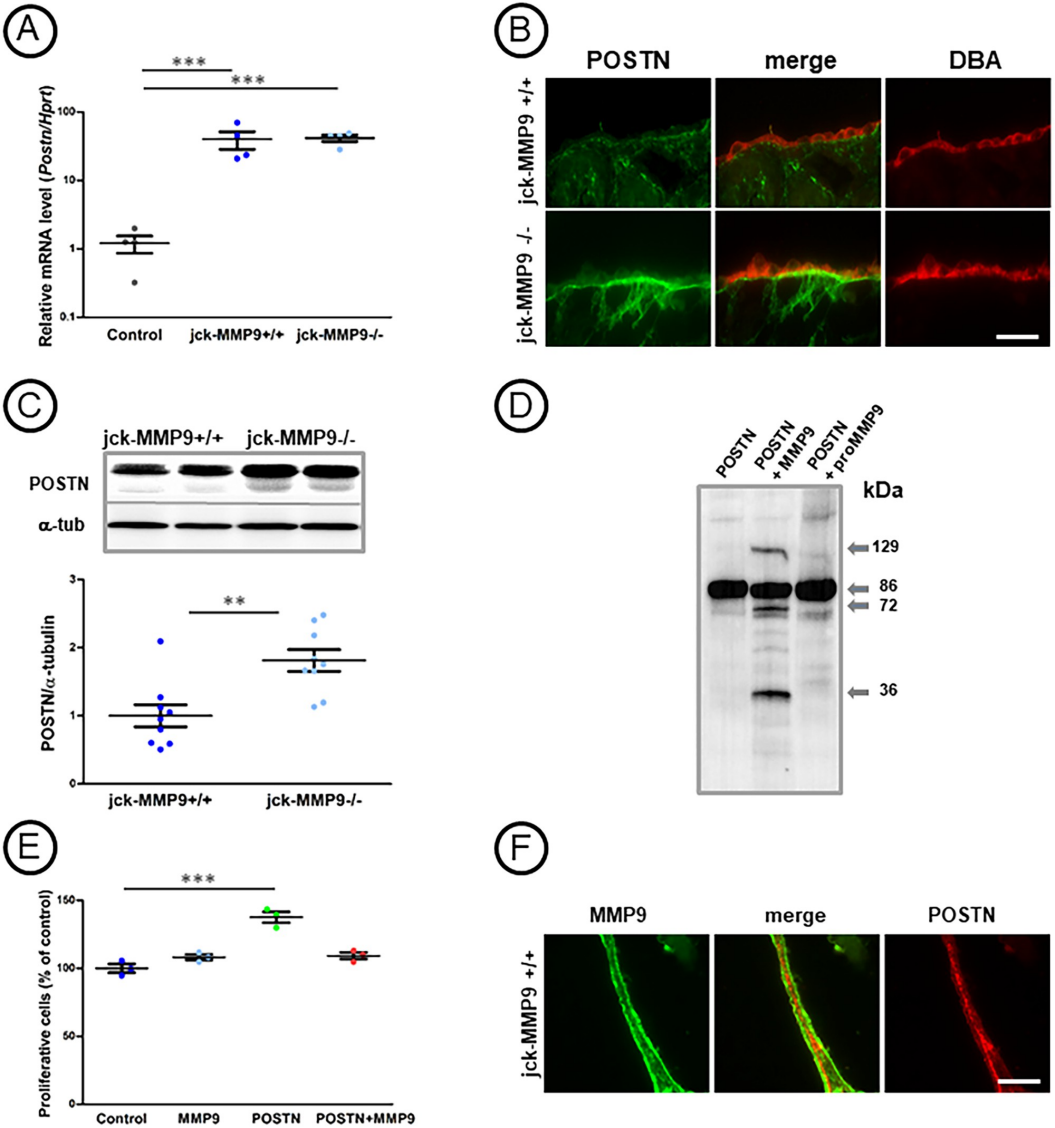
Effect of MMP9 on periostin expression. (A) Quantitative real time RT-PCR determination of *Periostin* (*Postn*) gene expression normalized to *Haptoglobin-related protein* (*Hptr*) in 4 control C57bl6, 4 jck-MMP9+/+ and 4 jck-MMP9-/- kidneys. Note that *Periostin* expression is significantly increased in jck kidneys compared to controls but is not modified in jck-MMP9+/+ versus jck-MMP9-/- kidneys. Values are mean ± SEM, ***p < 0.0001. (B, C) Comparative periostin protein (POSTN) expression in jck-MMP9+/+ and jck-MMP9-/- kidneys. (B) Microphotographs of representative paraffin kidney sections of cystic jck-MMP9+/+ and jck-MMP9-/- kidneys stained with periostin (POSTN) showing an increased expression of periostin in collecting ducts, stained with DBA lectin, of jck-MMP9-/- compared to jck-MMP9+/+ kidneys. (C) Representative Western Blot and quantitative analysis performed with proteins lysates from 6 jck-MMP9+/+ and 7 jck-MMP9-/- kidneys. Values are mean ± SEM, **p = 0.0026. (D) Representative western blot illustrating periostin cleavage by MMP9. Recombinant periostin (POSTN) (50 ng) was untreated (first lane, POSTN) or incubated with 500 ng of activated recombinant MMP9 (POSTN + MMP9). MMP9 induces appearance of proteolytic bands of periostin, while the proteolytic activity is not observed when MMP9 is not activated by APMA treatment (POSTN + proMMP9). (E) Percentage of proliferative IMCD-3 cells grown overnight in control culture medium supplemented with APMA (control), with activated recombinant mouse MMP9 (MMP9), with recombinant mouse periostin (POSTN), and with recombinant periostin preincubated with activated MMP9 (POSTN + MMP9). Note that cell proliferation is increased by POSTN by 50% but is identical in control cells and cells supplemented with POSTN (30 ng/ml) and MMP9 (500 ng/ml). Values are mean ± SEM, ***p < 0.0001. (F) Microphotographs of representative paraffin kidney sections of cystic jck-MMP9+/+ stained with periostin and MMP9 showing that both proteins are observed in the same tubules with an irregular co-localization. Scale bar: 100 μm.

Because periostin can increase MMP9 expression [21], but also aggravates cyst growth and fibrosis in pcy mouse cystic kidneys [22, 23], we first ask whether its expression could be modified in the Nek8^jck^ (jck) model. Indeed, we observed a boost of MMP9 enzymatic activity in this model of nephronophthisis (Fig 1A and 1B), and a ~40-fold increase of periostin mRNA by RT-qPCR. However, *Periostin* mRNA was similar in jck-MMP9 deficient and jck-control kidney (Fig 5A). We then ask whether periostin could be control by MMP9 at the protein level.

### Periostin is a new substrate of MMP9

To address this question, we compared periostin staining in cystic control (jck-MMP9-/-) and deficient (jck-MMP9+/+) kidneys, with a particular attention to collecting duct cells since MMP9 is expressed constitutively [20] in this nephron segment. Surprisingly, results obtained by immunofluorescence showed that MMP9 deficiency was associated with an intense staining of periostin at the basal side of the collecting duct cells (Fig 5B). Results were confirmed by western blotting where we could observe an increase level of periostin in kidney cell lysates of jck-MMP9-/- compared to jck-MMP9+/+ (Fig 5C). As these results strongly suggest that periostin could be a substrate of MMP9 in kidney, we performed *in vitro* experiments by incubating recombinant 6His-tagged MMP9 protein (500 ng) activated with APMA with recombinant periostin protein (50 ng). We indeed observed bands at 129, 72, and 36 kDa that appeared after 3 hours at 37°C. There were not seen when MMP9 was not activated with APMA (proMMP9), (Fig 5D). The upper band (129 kDa) was detected in 4 different experiments and could correspond to aggregated fragment of periostin or complex of activated MMP9 with periostin. As periostin is known to increase cell proliferation in ADPKD cyst epithelial cells [24], we estimated *in vitro* the physiological relevance of the periostin cleavage by MMP9 by quantifying the proliferation of IMCD-3 cell line, which do not produce MMP9 (S1A Fig). To that instance, we treated cells overnight with recombinant MMP9 (300 ng/ml) activated with AMPA, with recombinant periostin (30 ng/ml) and with periostin incubated for 2 hours with MMP9 (S1B Fig). In those conditions, the native form of periostin was decreased by 50% (S1C Fig). The percentage of proliferating cells was increased in cells incubated with periostin, but not in cells incubated with both periostin and MMP9, suggesting that a concentration of 15 ng/ml of periostin (corresponding to a 50% reduction) is not enough to induce proliferation of IMCD-3 cells. Indeed, an increase of cell proliferation by periostin has been described at 100 ng/ml in cystic epithelial cells [24] and 5 μg/ml in MIP101 colorectal cancer cells [25]. We next investigated the location of periostin and MMP9 in control cystic kidney (Fig 5F). Both proteins were observed in cells lining cysts where MMP9 was mostly located at the apical pole of the cells and periostin in the basal side. However, co-localization was sporadically observed (arrows) in the extracellular matrix suggesting that they are co-expressed after their secretion. Knowing that periostin protein level is increased in MMP9 deficient kidney compared to control cystic kidney, we next aimed to understand whether the accumulation of this protein contributes to the severity of the kidney disease observed in mice lacking MMP9. We first investigated the impact of an increase level of periostin on the amount of integrins.

### MMP9 deficiency increases integrins expression

**Fig 6:**
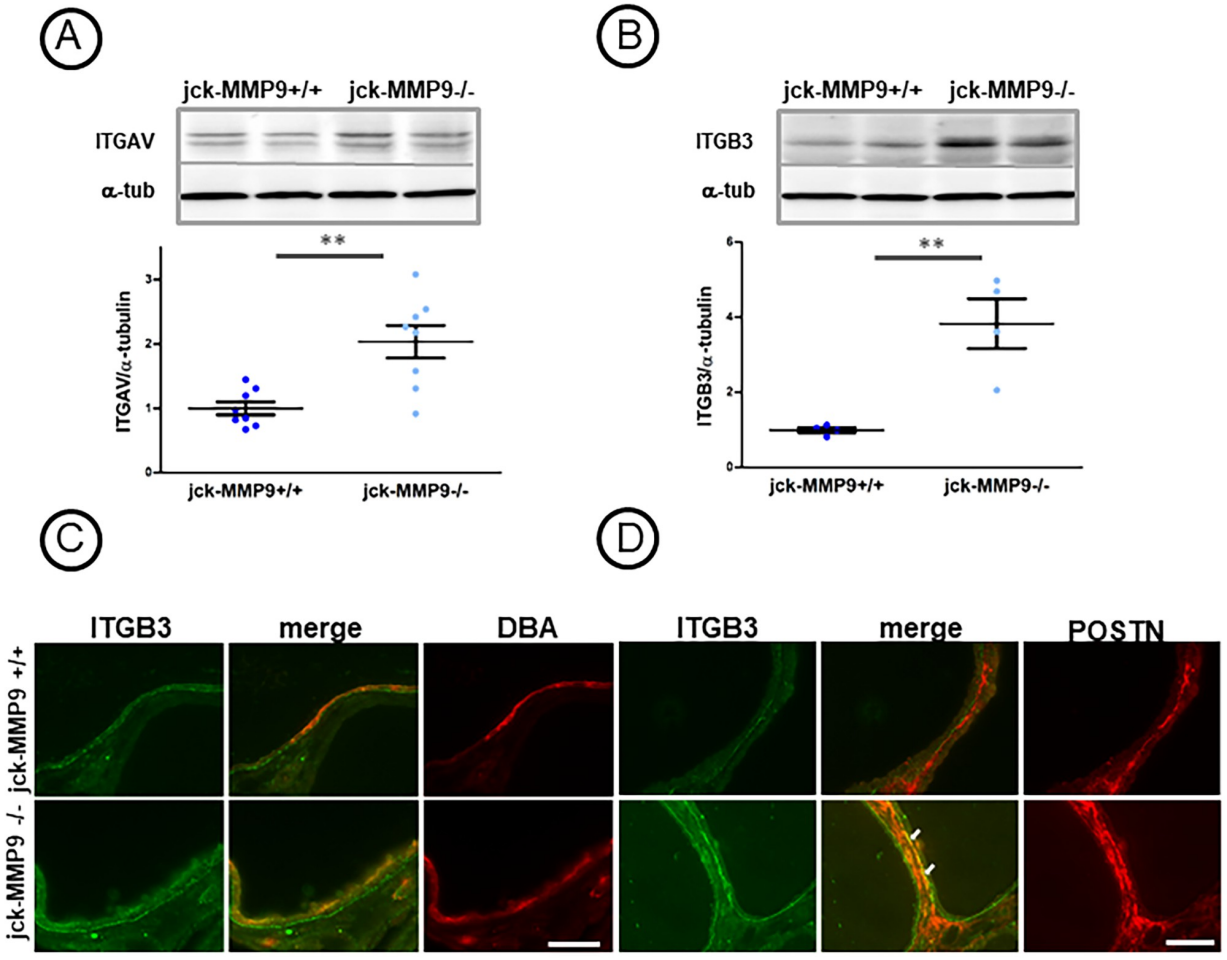
Effect of MMP9 on integrin expression. (A, B) Comparative integrins ITGAV (A) and ITGB3 (B) expression in jck-MMP9+/+ and jck-MMP9-/- kidneys by western blotting. Please note that expression of ITGAV and ITGB3 are significantly increased in jck-MMP9-/- kidneys as observed in representative western blots and quantitative analysis. Values are mean ± SEM, **p = 0.0019 and 0.0053, respectively. (C) Expression of ITGB3 in collecting duct cells stained with DBA of jck-MMP9+/+ and jck-MMP9-/- kidneys. (D) Expression of ITGB3 and POSTN in cystic tubules of 4 jck-MMP9+/+ and 4 jck-MMP9-/- kidneys. Please note co-expression of ITGB3 and periostin at the base of cystic tubules (arrow) and the increased expression of the co-staining in jck deficient kidneys (jck-MMP9-/-). Scale bar: 100 μm.

Dysregulation of integrin expression is a key feature of tissue remodeling during cyst formation [26]. Knowing that periostin is a ligand for αvβ3 integrin in ADPKD cells [24], we compared the expression of these integrins in control (jck-MMP9+/+) and MMP9 deficient (jck-MMP9-/-) kidneys. MMP9 deficiency increased the quantity of both αV (Fig 6A) and β3 (Fig 6B) integrins in kidney cell lysates observed by western blotting, and of β3 integrin in collecting ducts cells observed by immunofluorescence (Fig 6C). The β3 integrin co-localized with periostin at the base of the cyst (Fig 6D).

We also measured the amount of β1 integrin because it plays a role in cytogenesis [27, 28], and it could interact with periostin [29] and bind to [30] and be degraded by MMP9 [31]. Beta1 integrin intensity of staining was increased in the absence of MMP9 as demonstrated by western blotting (S2A Fig) and immunofluorescence where it was observed in collecting duct stained with the lectin DBA (S2B Fig). As a control, we compared the quantity of α3 integrin, which is neither a ligand of periostin nor a ligand of MMP9. Results in S3 Fig showed that it was not modified by western blotting or by immunofluorescence in jck-MMP9 -/- compared to jck-MMP9+/+ kidneys. These data show that MMP9 deficiency in cystic kidney is associated with an increase amount of both periostin and integrins.

### MMP9 inhibition increased FAK and ILK signaling pathways

**Fig 7:**
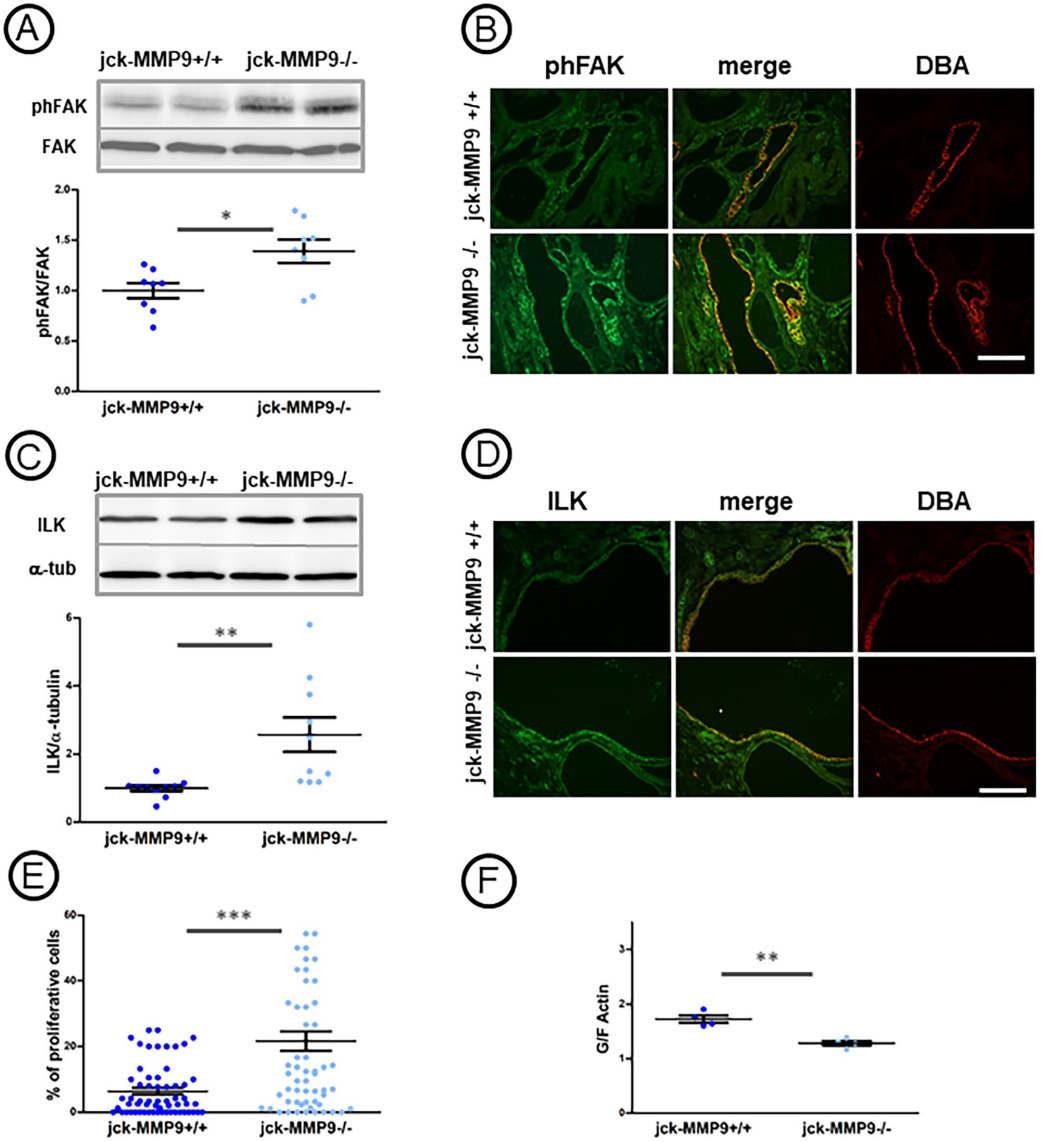
Effect of MMP9 on phFAK and ILK expression. (A, B) Comparative phFAK expression in jck-MMP9+/+ and jck-MMP9-/- kidneys. (A) Representative Western Blot and quantitative analysis performed with proteins lysates from 8 jck-MMP9+/+ and 8 jck-MMP9-/- kidneys. Values are mean ± SEM, **p = 0.0140. (B) Microphotographs of representative paraffin kidney sections of cystic jck-MMP9+/+ and jck-MMP9-/- kidneys stained with phFAK and DBA lectin. The expression of phFAK in collecting ducts stained with DBA is increased in jck-MMP9-/- compared to jck-MMP9+/+ kidneys. (C, D) Comparative ILK expression in jck-MMP9+/+ and jck-MMP9-/- kidneys by western blotting (C) and immunofluorescence (D). Please note that expression of ILK is significantly increased in jck-MMP9-/- compared to jck-MMP9+/+ kidneys as observed in representative western blots and quantitative analysis. Values are mean ± SEM, **p = 0.0066. (E) Percentage of proliferative cells in collecting duct cells of jck-MMP9+/+ and jck-MMP9-/- kidneys. The percentage of proliferative cells is dramatically increased upon MMP9 deficiency in cystic kidney. Values are mean ± SEM, ***p < 0.0001 (F) G/F actin ratio in 4 jck-MMP9+/+ and 4 jck-MMP9-/- kidney cell lysates. Note that the ratio is significantly decreased upon MMP9 deficiency. Values are mean ± SEM, ***p = 0.0016. Scale bar: 100 μm.

Binding of integrins to extracellular matrix ligands lead to formation of focal adhesions. They are composed of multiprotein complexes and the activation of integrins is followed by recruitment of two major kinases, integrin-linked kinase (ILK) and focal adhesion kinase (FAK) [32]. ILK interacts with β1 and β3 integrins [33] and FAK with β1 integrin [34]. To determine whether integrins dysregulation resulting to MMP9 inhibition could activate signaling pathways such as FAK and ILK, we performed western blotting and immunofluorescent studies on jck-MMP9+/+ and jck-MMP9-/- kidneys. Results showed an increase level of phosphorylation of FAK at the Y397 site and ILK expression in lysates (Fig 7A and 7C, respectively) and in collecting ducts cells (Fig 7B and 7D, respectively) of jck-MMP9-/- compared to jck-MMP9+/+ kidneys. Therefore, the increase of the integrin level in jck-MMP9-/- kidneys is associated with an enhance activation of the major kinases of the focal adhesion, ILK and FAK.

### MMP9 deficiency do not modify cell apoptosis but increases cell proliferation and polymerization of actin cytoskeleton

The kinases FAK and ILK are essential intermediate between ECM/integrin and the actin cytoskeleton [33, 35]. We have shown that MMP9 protects from apoptosis [13], and it is known that both ILK and activation of FAK regulate cell proliferation in polycystic kidney disease [26]. For these reasons, we estimated the percentage of apoptotic cells by TUNEL method and of proliferative cells with PCNA staining. Apoptosis did not differ between jck-MMP9+/+ and MMP9-/- mice during the course of the study (S4 Fig). However, the cell proliferation, assessed by the number of nuclei of collecting duct cells stained with PCNA, was increased in jck-MMP9-/- compared to control kidneys (Fig 7E). ILK and phosphorylation of FAK also promotes actin polymerization [35, 36]. Accordingly, we ask whether MMP9 could control actin dynamics. To that instance, we evaluate the actin polymerization by comparing the ratio of globular (G)/ filamentous (F) actin in control and MMP9 deficient jck kidney. Indeed, we confirmed that MMP9 deficiency is associated with an increase of filamentous actin assessed by a decreased G/F ratio (Fig 7F). These results suggest that MMP9 deficiency contributes to deteriorate renal function and exacerbate renal lesions by interfering with cell proliferation and cytoskeletal actin organization.

## Discussion

In the present study, we observed an increased activity of MMP9 in kidney tissue of jck mice. Comparison of jck-MMP9+/+ and jck-MMP9-/- mice led us to show a protective role of MMP9 in the pathology of polycystic kidney disease and to uncover a new substrate of MMP9.

MMP9 deficient mice exhibit a 30% decrease in nephron number. However, kidney function and morphology and immunohistochemical analysis of several basement membrane components [37] are normal up to 12 months where they develop interstitial fibrosis but their life span still remained identical at that age [12]. To specify the role of MMP9 in cystic disease, we bred jck (juvenile cystic kidneys) mice [18] with MMP9 deficient mice [19]. Because genetic background considerably influences the manifestations of the disease in different mouse models of PKD including jck [38, 39], both strains of mice were derived on the same C57bl/6J genetic background and several backcross breedings were performed to obtain inbred double mutant mice. Jck mouse model results from a double point mutation in the *Nek8* gene [18]. While mutations in this gene are responsible for nephronophthisis in humans [40], mutant mice display features that recapitulate an ADPKD phenotype [41, 42]. The disease progresses relatively rapidly to end stage renal disease at 4 months. Cysts originate from the collecting ducts, the loop of Henle and distal tubules [43]. Our data show that MMP9 protects jck kidney from cystic phenotype. MMP9 expression and activity in cystic jck kidneys compared to controls was noticeably increased. MMP9 was observed in cells lining cystic collecting ducts. Although MMP9 staining was observed exclusively at the apical pole of control collecting ducts cells [20], staining extended to cytoplasm in cells lining cystic collecting ducts. Interestingly, similar results were found in human ARPKD [44]. The effect of MMP9 is beneficial as the cystic index, the kidney weight and the number of cysts were increased in MMP9 deficient kidneys. Previous studies concerning the role of metalloproteinases in cystic disease were unclear. They showed that a broad inhibition of MMPs accelerates cyst growth in pcy mice [9] while it reduces the percentage of cystic tissue in pck rat model [10] and the number of cysts in the cy/+ rat model [11]. These discrepant findings might be explained not only by the different genetic background between rat and mice but also by the disparity of the cystic models. In addition, MMPs are broadly inhibited in these models and the particular role and expression of a distinct metalloproteinase is unspecified. In our model, the specific role of MMP9 on cyst formation is suggested by the steady levels of MMP2 (Fig 1A). By contrast, MMP2 expression and activity compared to MMP9 are increased in cpk cystic mouse model. The prominent localization of MMP2 in this model was in the interstitium and in foci between the cysts [7], suggesting a role in the control of fibrosis rather than of cyst enlargement. In addition to the aggravated cystic phenotype, the percentage of fibrosis was noticeably increased in MMP9 deficient mice. Because the extent of fibrosis is strongly associated with the rate of progression to end stage renal disease in ADPKD patients [45], this result could account for the protective role of MMP9 in the pathology, as demonstrated by improved kidney function and life expectancy. To understand the MMP9 protection mechanism in this model, we search for proteins that could control or be controlled by MMP9. To that intent, we focused on periostin that increase MMP9 expression in the CKD model of 5/6 nephrectomy [21] and on β1 integrin that is cleaved by MMP9 [31].

Periostin is a matricellular protein, which is *de novo* expressed during the development of renal disease and is a target of therapy in chronic renal disease (CKD) [46]. As previously shown *in vitro* in human ADPKD cell [24] and *in vivo* in the pcy murine model of nephronophthisis [23], we also observed an overexpression of periostin in epithelial cells lining the cysts in the jck murine model. However, while periostin mRNA were similar in jck control et MMP9 deficient mice, its protein level was increased in jck-MMP9 -/- mice by immunofluorescence and western blotting. We therefore hypothesize that this protein could be a substrate of MMP9, and showed that indeed MMP9 could cleave this protein *in vitro* and could reduce its physiological function such as proliferation. Proteomic approach in the human vasculature identified periostin as a substrate of MMP3 and MMP14 but the degradation by MMP9 was not statistically significant [47], suggesting that the sensitivity to MMP9 cleavage of periostin could be tissue specific. The increased periostin level in jck-MMP9 -/- mice could *per se* explain the severity of their phenotype since selective overexpression of periostin in collecting duct cells of pcy mice aggravates their cystic phenotype [22]. Conversely, pcy mice deficient for periostin are protected and exhibit a decrease kidney mass and cystic index, a reduced extent of fibrosis and increase life span [23].

We investigated the expression of integrins, which are the receptors of both periostin and MMP9. β1 integrin has a special interest because it could interact with periostin [29], and it is required for renal cytogenesis caused by ciliary defect [27] and for development of ADPKD after the loss of polycystin-1 [28]. In addition, Integrin β1 both associates [30] and is cleaved by MMP9 [31]. The increase of β1 integrin protein content in cystic MMP9 deficient mice is associated with worsening of the phenotype and is in favor of a regulation of this integrin by MMP9. We next evaluated the amount of αvβ3 integrin, which is along with αvβ5 a receptor of periostin [24]. As observed in ADPKD cells *in vitro*, increase periostin level in cystic MMP9 deficient mice is related to higher amounts of αv and β3 integrins, of ILK and of FAK phosphorylation at Tyr397 [22]. Inactivation of ILK in collecting ducts of PKHD1 or pcy mice models reduced cell proliferation, cystic development and fibrosis and extends the life of the mice [48]. Similarly, cell proliferation was decrease following inhibition of FAK activity *in vitro* and *ex vivo* in 3D MDCK cell culture model. In PKD1 knock out mouse models, FAK inhibitors delay cyst formation and improve renal function [49]. After injury of retinal ganglion cell, MMP9 is increased and FAK activation is decreased. In this model, FAK activation induced cell survival despite MMP9 activation [50]. Because β1 integrin, ILK and activated FAK interact with cytoskeleton at focal contact and associates with the actin filament binding molecules [26, 35, 36], we assessed the G/F actin ratio and observed that actin filaments are increased upon MMP9 inhibition in the jck mouse model.

In conclusion, we provide evidence that MMP9 is overexpressed in cystic tissue of the jck mouse model and exerts a protective effect on cyst formation, fibrosis and consequently increase the lifespan of the mice. In addition, we uncover a new substrate of MMP9, periostin. MMP9 deficiency in the jck model is associate with higher content of periostin and β1 integrin, a known MMP9 substrate [30, 31]. In turn, periostin level correlates with the increase of other integrins such as αv and β3 integrins. Overall, the augmentation of integrins activate FAK and ILK intracellular signaling pathway leading to cell proliferation and cytoskeletal actin polymerization. These effects could account for the control of the renal function, the cyst formation and the fibrosis extent of the mice.

## Supporting information

S1 Fig Physiological effect of cleavage of periostin by MMP9 on IMCD-3 cell proliferation. (A) Representative zymography of control IMCD-3 cells and cells treated with periostin, activated MMP9 and periostin + activated MMP9. Note that IMCD-3 cell line does not express MMP9. (B, C) Representative Western Blot and quantitative analysis showing that periostin level is decreased by 50% after incubation with activated MMP9 overnight. (D) Microphotographs of representative IMCD-3 cells stained with Ki67 conjugated to eFluor^™^ 660 and DAPI showing an increased level of cell proliferation in cells treated with periostin but not in cells treated with periostin and MMP9. Scale bar: 50 μm.
(TIF)

S2 Fig Effect of MMP9 on the expression of β1 integrin (ITGB1) in control (jck-MMP9+/+) and MMP9 deficient (jck-MMP9-/-) jck kidneys. (A) Representative Western Blot and quantitative analysis performed with proteins lysates from 16 jck-MMP9+/+ and 14 jck-MMP9-/- kidneys. Note the significant increased expression of ITGB1 normalized to a-tubulin in jck-MMP9-/- mice. Values are mean ± SEM, **p = 0.0061. (B) Microphotographs of representative paraffin kidney sections of cystic jck-MMP9+/+ and jck-MMP9-/- kidneys stained with IGTB1 showing an increased expression of IGTB1 in cystic collecting ducts, stained with DBA lectin, of jck-MMP9-/- compared to jck-MMP9+/+ kidneys. Scale bar: 100 μm.
(TIF)

S3 Fig Effect of MMP9 on the expression of α3 integrin (ITGA3) in control (jck-MMP9+/+) and MMP9 deficient (jck-MMP9-/-) jck kidneys. Please note that expression of ITGA3 is not modified in jck-MMP9-/- kidneys as observed in representative pictures (A) and quantitative analysis of western blots (B). Scale bar: 100 μm.
(TIF)

S4 Fig Effect of MMP9 on the percentage of apoptotic collecting ducts cells in jck kidneys at 1 month and 4 months after birth. The percentage was determined on 6 micrographs taken from 8 different kidneys of each group at 1 month and at 4 months.
(TIF)

S1 Raw images (PDF)
