# Supplementary figures and images for: Cleavage of periostin by MMP9 protects mice from kidney cystic disease
Source: PLoS One. 2023 Dec 1;18(12):e0294922. doi: 10.1371/journal.pone.0294922 (PMC10691688; doi:10.1371/journal.pone.0294922)

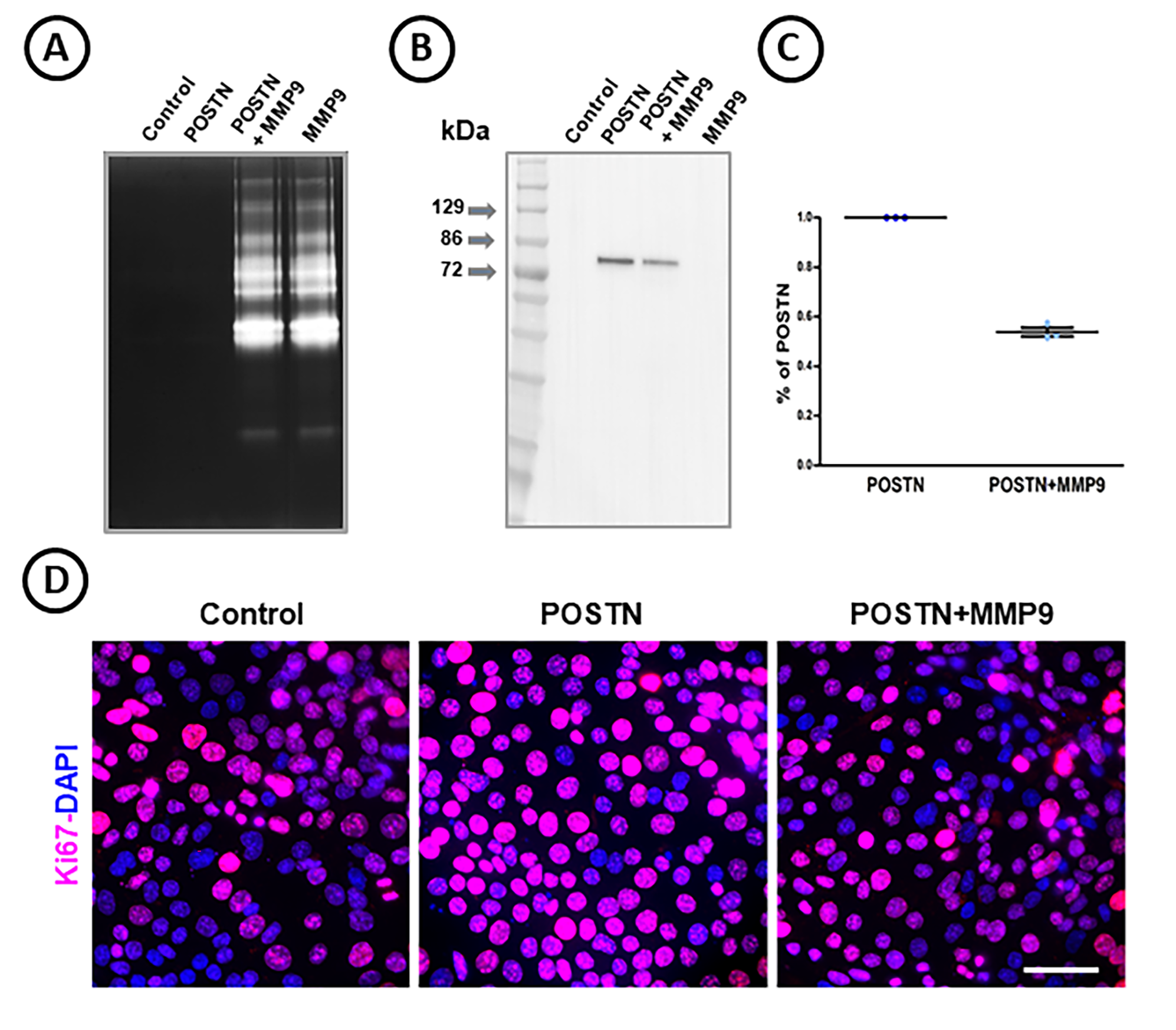

Supplement: S1 Fig — (A) Representative zymography of control IMCD-3 cells and cells treated with periostin, activated MMP9 and periostin + activated MMP9. Note that IMCD-3 cell line does not express MMP9. (B, C) Representative Western Blot and quantitative analysis showing that periostin level is decreased by 50% after incubation with activated MMP9 overnight. (D) Microphotographs of representative IMCD-3 cells stained with Ki67 conjugated to eFluor™ 660 and DAPI showing an increased level of cell proliferation in cells treated with periostin but not in cells treated with periostin and MMP9. Scale bar: 50 μm. (TIF) [file pone.0294922.s001.tif]

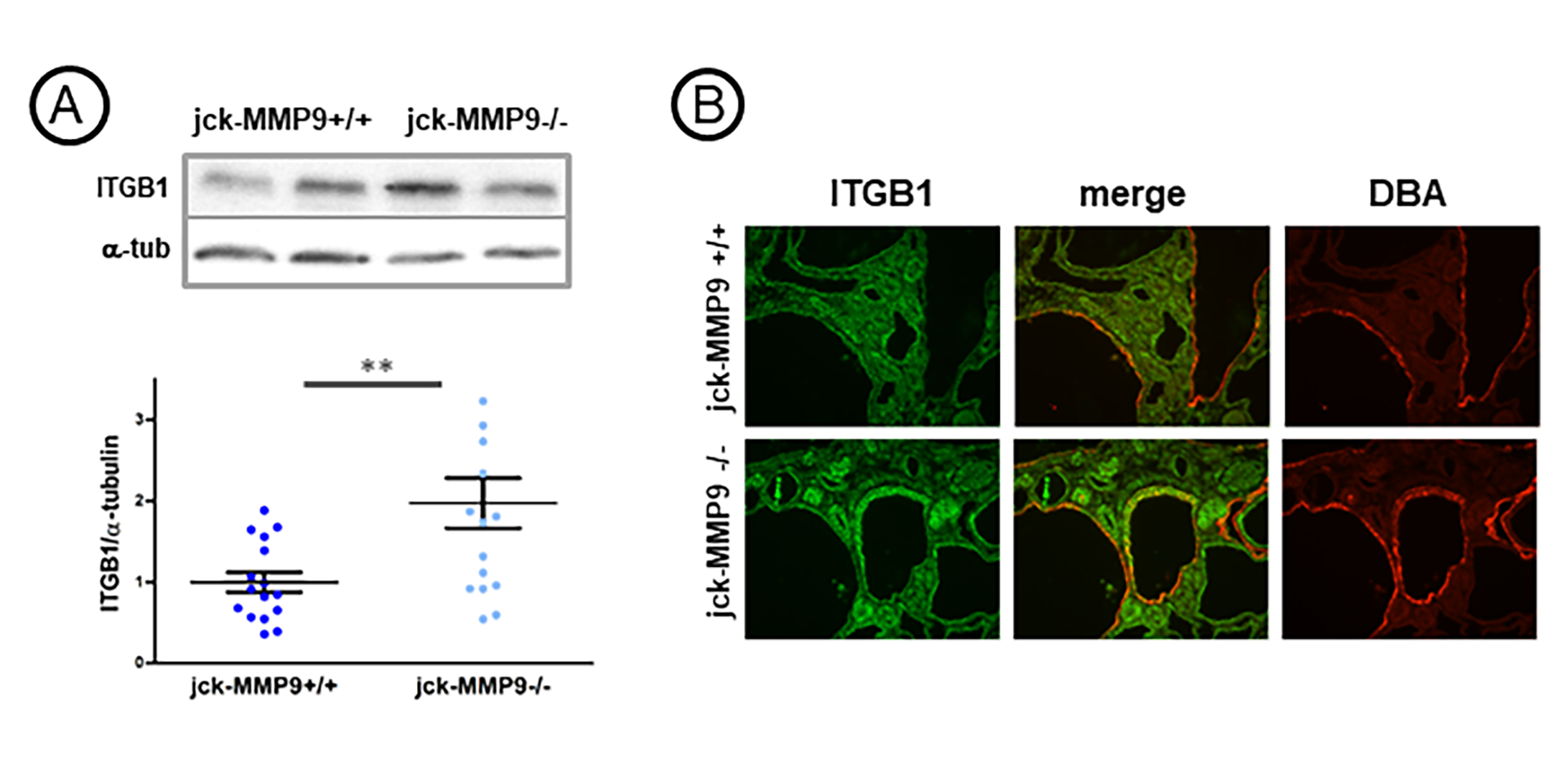

Supplement: S2 Fig — (A) Representative Western Blot and quantitative analysis performed with proteins lysates from 16 jck-MMP9+/+ and 14 jck-MMP9-/- kidneys. Note the significant increased expression of ITGB1 normalized to a-tubulin in jck-MMP9-/- mice. Values are mean ± SEM, **p = 0.0061. (B) Microphotographs of representative paraffin kidney sections of cystic jck-MMP9+/+ and jck-MMP9-/- kidneys stained with IGTB1 showing an increased expression of IGTB1 in cystic collecting ducts, stained with DBA lectin, of jck-MMP9-/- compared to jck-MMP9+/+ kidneys. Scale bar: 100 μm. (TIF) [file pone.0294922.s002.tif]

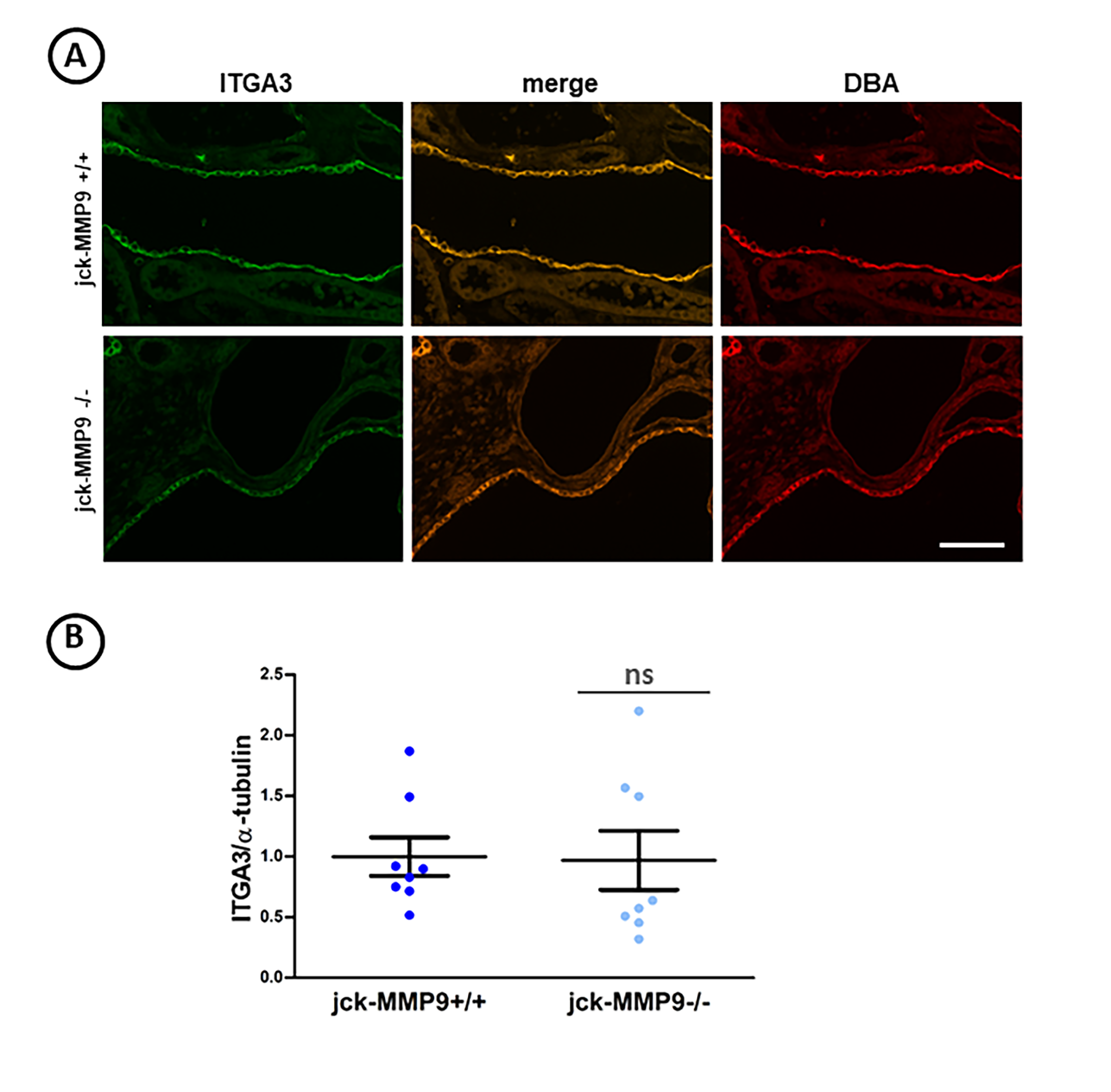

Supplement: S3 Fig — Please note that expression of ITGA3 is not modified in jck-MMP9-/- kidneys as observed in representative pictures (A) and quantitative analysis of western blots (B). Scale bar: 100 μm. (TIF) [file pone.0294922.s003.tif]

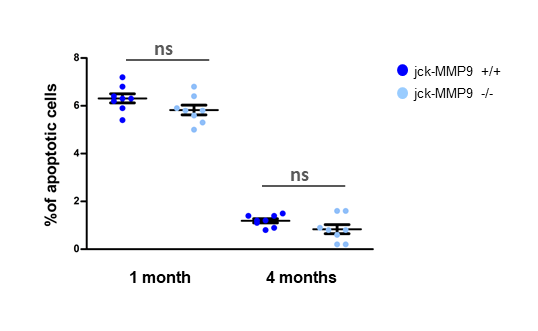

Supplement: S4 Fig — The percentage was determined on 6 micrographs taken from 8 different kidneys of each group at 1 month and at 4 months. (TIF) [file pone.0294922.s004.tif]
