# Supplementary material for: Cleavage of periostin by MMP9 protects mice from kidney cystic disease
Source: PLoS One. 2023 Dec 1;18(12):e0294922. doi: 10.1371/journal.pone.0294922 (PMC10691688; doi:10.1371/journal.pone.0294922)
Supplement: S1 Raw images — (PDF) [file pone.0294922.s005.pdf]

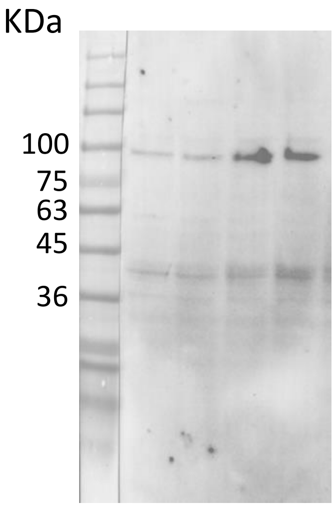

Figure 1B

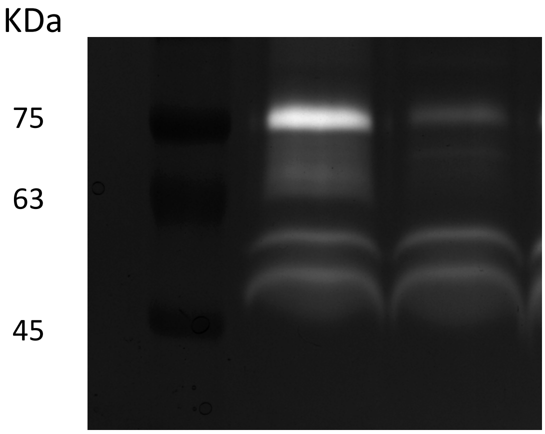

Figure 1C

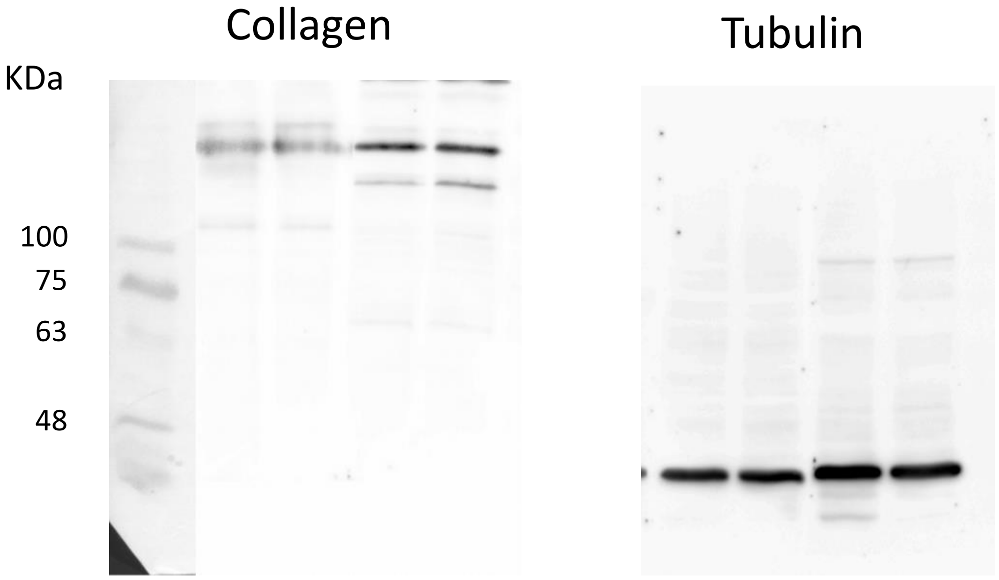

Figure 4C

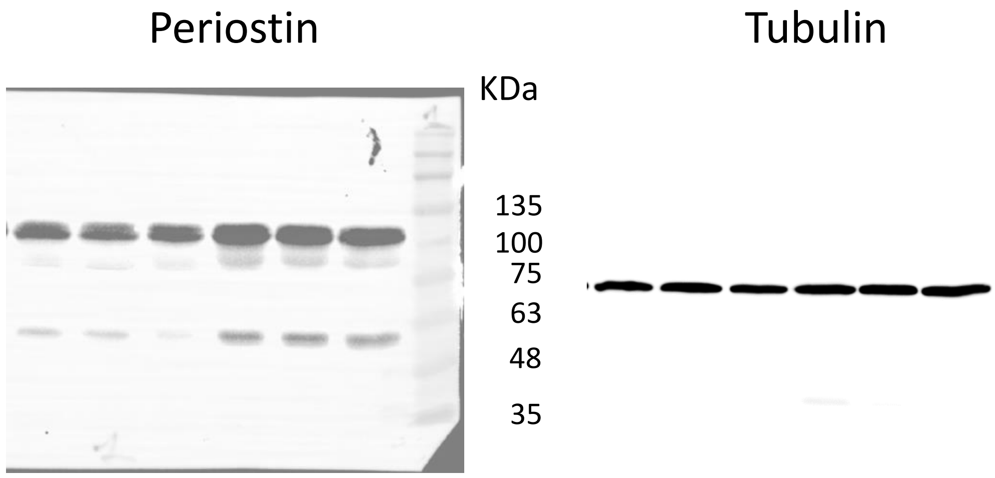

Figure 5C

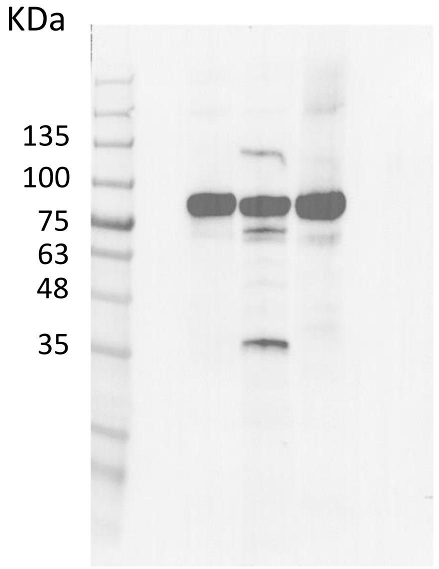

Figure 5D

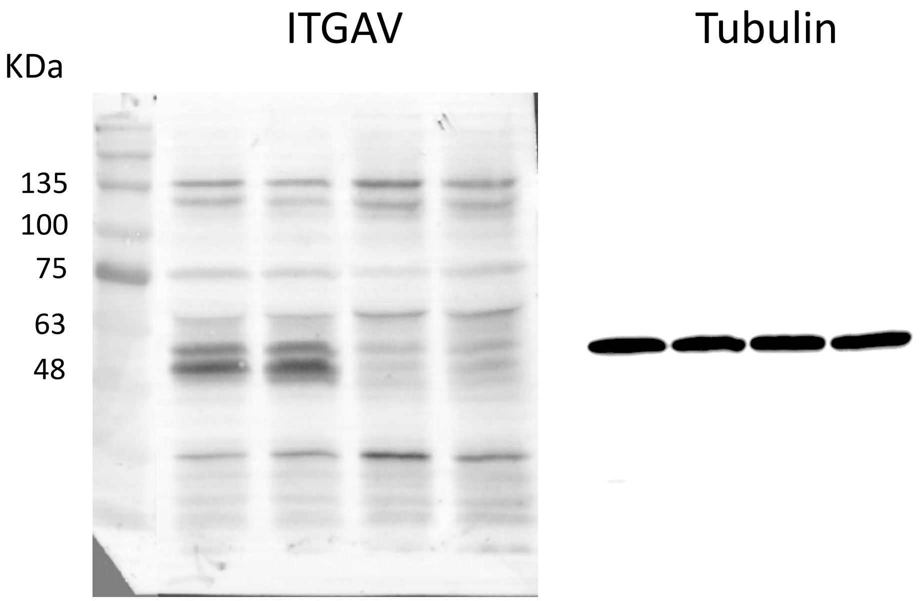

Figure 6A

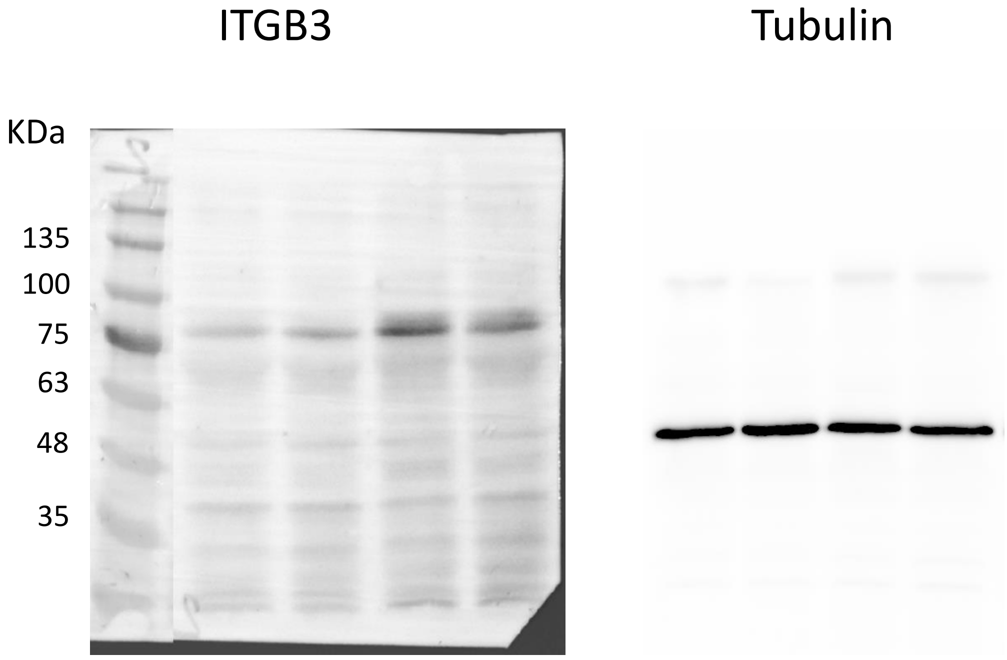

Figure 6B

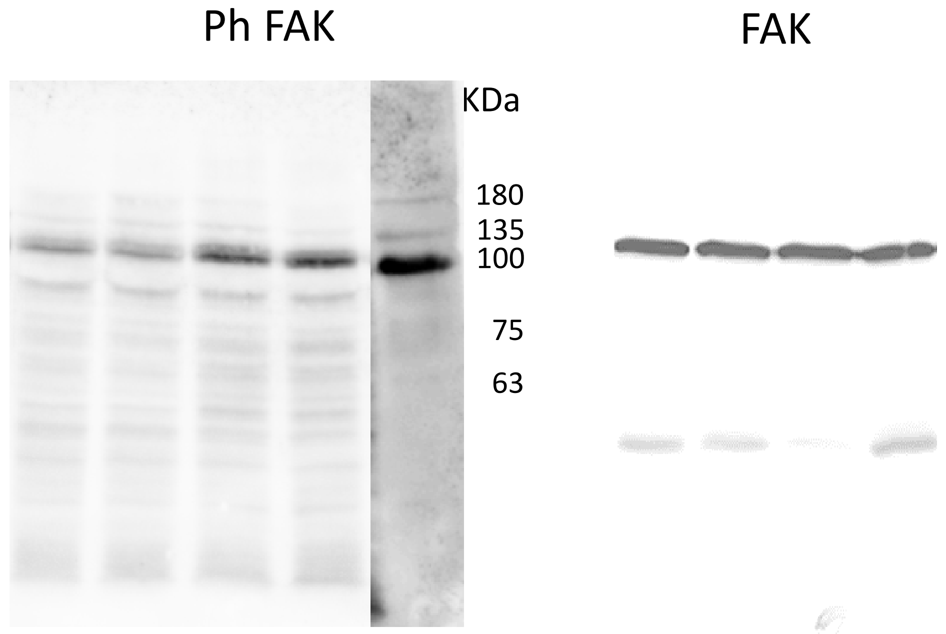

Figure 7A

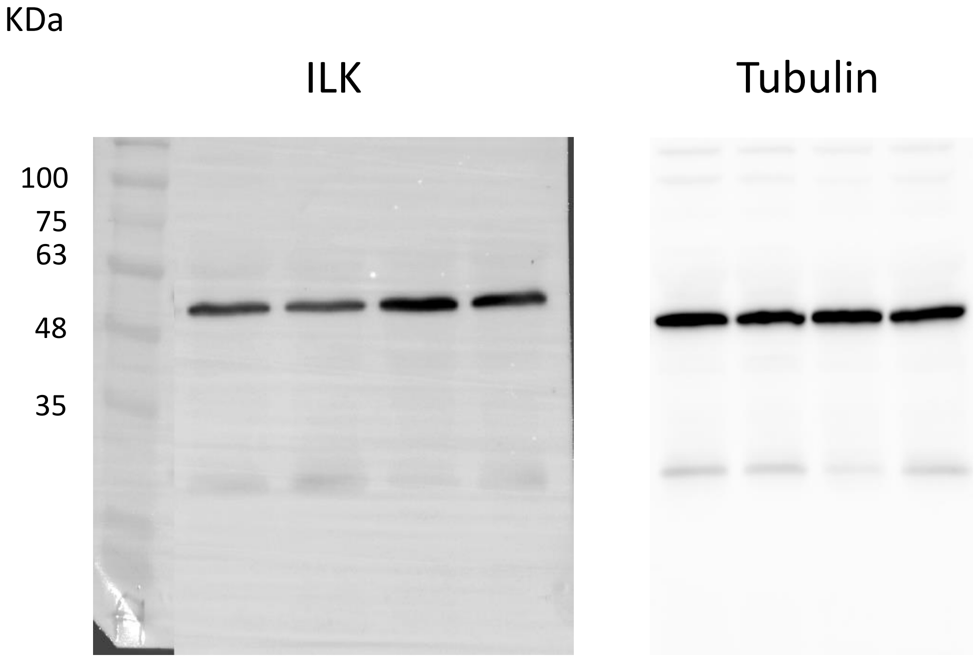

Figure 7C

Zymography

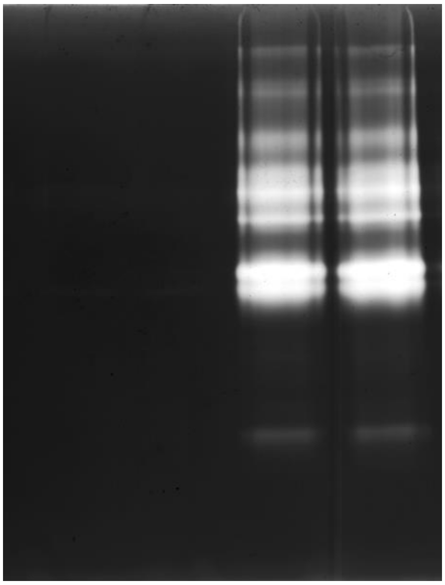

POSTN

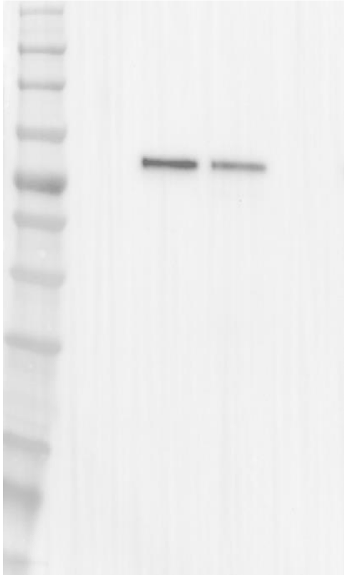

S21Fig

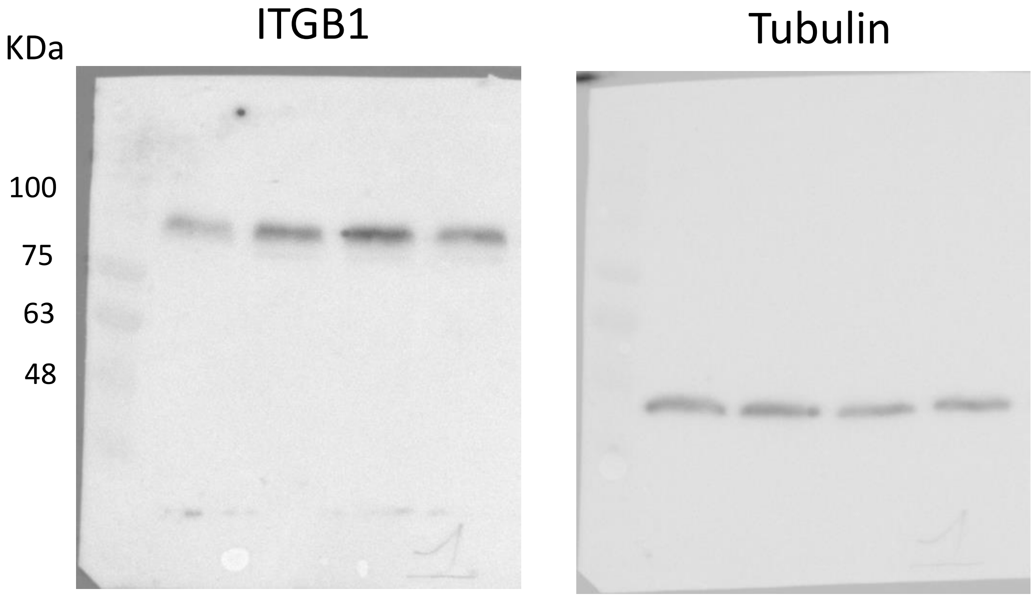

S2 Fig
